# Understanding spatiotemporal symptom onset risk of Omicron BA.1, BA.2 and hamster-related Delta AY.127

**DOI:** 10.3389/fpubh.2022.978052

**Published:** 2022-09-16

**Authors:** Chengzhuo Tong, Wenzhong Shi, Gilman Kit-Hang Siu, Anshu Zhang, Zhicheng Shi

**Affiliations:** ^1^Department of Land Surveying and Geo-Informatics, Otto Poon Charitable Foundation Smart Cities Research Institute, The Hong Kong Polytechnic University, Kowloon, Hong Kong SAR, China; ^2^Department of Health Technology and Informatics, The Hong Kong Polytechnic University, Kowloon, Hong Kong SAR, China; ^3^Research Institute for Smart Cities, School of Architecture and Urban Planning, Shenzhen University, Shenzhen, China

**Keywords:** Omicron BA.1 and BA.2, hamster-related Delta AY.127, spatiotemporal symptom onset risk, full vaccination, booster vaccination

## Abstract

**Purpose:**

Investigation of the community-level symptomatic onset risk regarding severe acute respiratory syndrome coronavirus 2 (SARS-CoV-2) variants of concern, is crucial to the pandemic control in the new normal.

**Methods:**

Investigated in this study is the spatiotemporal symptom onset risk with Omicron BA.1, BA.2, and hamster-related Delta AY.127 by a joint analysis of community-based human mobility, virus genomes, and vaccinations in Hong Kong.

**Results:**

The spatial spread of Omicron BA.2 was found to be 2.91 times and 2.56 times faster than that of Omicron BA.1 and Delta AY.127. Identified has been an early spatial invasion process in which spatiotemporal symptom onset risk was associated with intercommunity and cross-community human mobility of a dominant source location, especially regarding enhancement of the effects of the increased intrinsic transmissibility of Omicron BA.2. Further explored is the spread of Omicron BA.1, BA.2, and Delta AY.127 under different full and booster vaccination rate levels. An increase in full vaccination rates has primarily contributed to the reduction in areas within lower onset risk. An increase in the booster vaccination rate can promote a reduction in those areas within higher onset risk.

**Conclusions:**

This study has provided a comprehensive investigation concerning the spatiotemporal symptom onset risk of Omicron BA.1, BA.2, and hamster-related Delta AY.127, and as such can contribute some help to countries and regions regarding the prevention of the emergence of such as these variants, on a strategic basis. Moreover, this study provides scientifically derived findings on the impact of full and booster vaccination campaigns working in the area of the reduction of symptomatic infections.

## Introduction

Designated as a variant of concern by the World Health Organization (WHO) on November 26th 2021 (1), the Omicron variant has become the dominant variant circulating globally (2). Currently, the sub-variants of Omicron are mainly divided into five groups: BA.1, BA.2, BA.3, BA.4, and BA.5 (3, 4). Of these, the BA.1 subvariant was initially the predominant of the global Omicron lineage, but the global proportion of COVID-19 cases, associated with the BA.2 variant had been increasing rapidly (5–8). Hong Kong had generally well controlled the attack of all former prominent COVID-19 variants, but resultantly the people had acquired little immunity from infections. During the global Omicron wave, Hong Kong became one of the global cities that suffered the most from BA.1 and BA.2 variants one after another (9). The BA.2 sub-group in Hong Kong, in particular, is unique when compared to variants, seen in the rest of the world due to unique mutations found in ORF1a: A2909V and ORF3a: L140F (10). It is also of note that, at the same time, an unusual cluster of probable hamster-to-human SARS-CoV-2 transmission due to the SARS-CoV-2 AY.127 variant has appeared in Hong Kong (11). Hong Kong's COVID-19 case fatality rate was one of the highest in the world (12), at the peak of the previous wave of outbreaks. Faced with a surge in cases during the previous epidemic wave, Hong Kong and other regions, fearing a more severe development of the disease, focused on prevention, and the early investigation of symptomatic cases (13). This is, now, currently the aim and practice of other countries and regions of the world, which now have likewise, rapidly aimed to prevent the worsening cases and deaths regarding the latest epidemic wave of COVID-19 (14, 15). To achieve early detection and treatment of symptomatic cases, it is necessary to effectively monitor and predict the risk level of symptomatic cases at the community level, the latter now becoming the focus of current epidemic prevention work in various countries (16–18).

Thus, in order to support more effective control of the spread of SARS-CoV-2 sub-lineages in such as Hong Kong and other countries, it is worthwhile to effectively predict and understand the spatiotemporal symptom onset risk for Omicron BA.1, BA.2, and hamster-related Delta AY.127 during the early spread stages. More importantly, the impact of COVID-19 vaccination, at the time of spatiotemporal symptom onset risk with Omicron BA.1, BA.2, and Delta AY.127 would provide benefits, if further explored (19).

Omicron BA.1 was detected for the first time, in virus gene sequencing samples from local airline pilots in Hong Kong as early as December 28th, 2021 (20). After that, BA.1 spread rapidly within the territory and, in fact, became a dominant variant until mid-January 2022. On January 16th, 2022, BA.2 was first detected in a virus sample from a local case entering the community after being infected during quarantine. It replaced BA.1 as the current dominant virus strain in Hong Kong, for a very short period, as the current dominant virus strain in Hong Kong. During this period, beginning on January 17th, 2022, the Delta SARS-CoV-2 AY.127 variants were continuously detected in virus samples from a cohort of employees and customers of a hamster pet store (21). During the early stages of the spread of these variants in Hong Kong, multipronged measures were taken to increase the uptake and pace of the vaccination, in terms of the inactivated (Sinovac) or mRNA (BioNTech) COVID-19 vaccine (22). From mid-December 2021 to early February 2022, the full vaccination rate in Hong Kong was increased from 61.3 to 64.3%, and the booster vaccination rate was increased from 3.7 to 12.8% (23).

**Figure 1:**
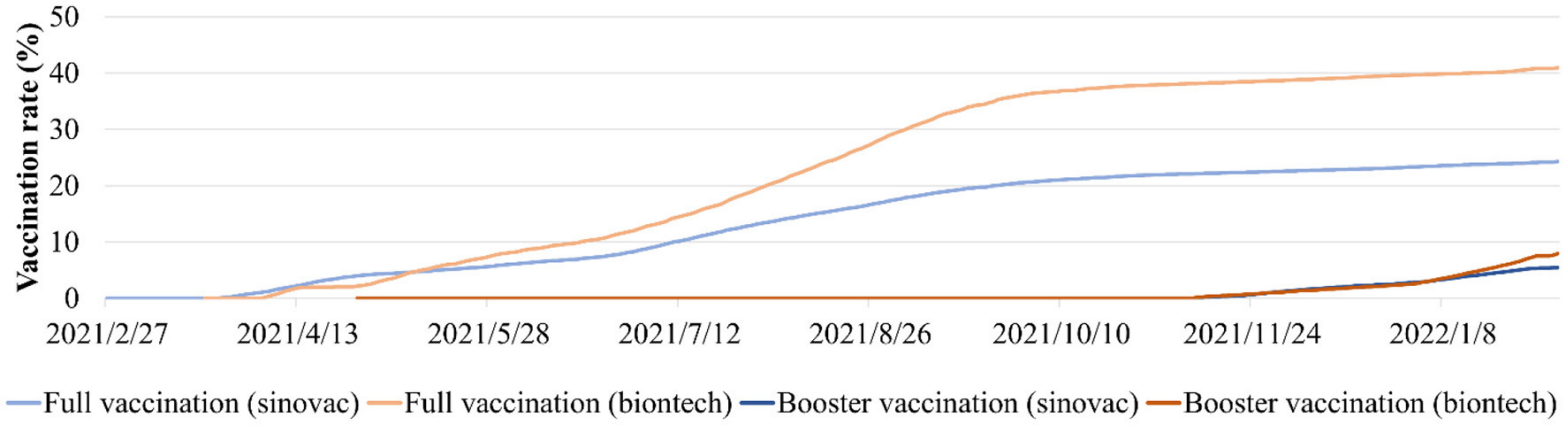
The daily full and booster variation rates of Sinovac and BioNTech vaccine in Hong Kong from February 22nd, 2021 to February 4th, 2022.

Thus, in order to understand the spatiotemporal spread of Omicron BA.1, BA.2, and Delta AY.127 in Hong Kong, firstly determined will be the SARS-CoV-2 sequences in these cases by whole-genome sequencing of respiratory specimens or deep throat saliva from the above cases, to determine the possible transmission linkage based on their phylogenetic relatedness (24–27). By the identified sequences and epidemiological link of these cases, the enhanced urban-community-level weighted kernel density estimation (WKDE) model (28–31) will then, be proposed to predict the spatiotemporal COVID-19 symptom onset risk of Omicron BA.1, BA.2, and Delta AY.127 in 291 Tertiary Planning Units (TPUs) of Hong Kong (Figure 1). (i) Locations with symptomatic cases resided/visited, (ii) locations with positive sewage testing results, (iii) time-varying vaccination rate and vaccination efficiency (6, 32–36) were incorporated to enhance the WKDE model. Based on the onset risk prediction results during the first 20 days, simulated were the early spatiotemporal spread of Omicron BA.1, BA.2, and Delta AY.127 under different scenarios and with different full and booster vaccination rate levels. The spatiotemporal data of the daily symptom onset cases in 291 TPUs of Hong Kong from December 26th, 2021, to February 4th, 2022, are utilized regarding the development of this study.

## Methods

### Data sources

This current study, involved the full genome sequencing and thereby concerned respiratory specimens or deep throat saliva from laboratory-confirmed COVID-19 patients admitted to both the main hospital, and the temporary hospital adjacent to the community treatment facility in Hong Kong. To enable the whole viral genome sequencing of patients' specimens to be conducted, the PCR tiling of the SARS-CoV-2 virus with rapid barcoding protocol was used (Version: mrt_9127_v110_revH_14Jul2021) on Nanopore GridION MK1 (Oxford Nanopore Technologies) (37). Between Dec 2021 and Feb 2022, a total of 652 cases were reported in Hong Kong. Because of a high number of reported cases, only the symptomatic cases were admitted to hospitals for treatment, whereas asymptomatic cases were mostly quarantined at home. We recruited all the positive cases from five public hospitals during that period for whole viral genome analysis. A total of 573 cases was collected and sequenced, which account for 87.9% of the total cases during the study period. Throughout the whole-genome sequencing process, data on i) 34 local cases with Omicron BA.1, ii) 231 local cases with Omicron BA.2, and iii) 16 local cases with Delta AY.127 during the period from December 2021 to February 2022, along with spatiotemporal information, were used in this study. Transmission clusters were defined by clear epidemiological linkage and onset-time relationship (27). The detail of local cases and transmission clusters used in this study have been made public in the Next Strain dataset (38). In addition, location data collected from sewage sample(s) tested positive during the period from December 2021 to February 2022 has also been used in this study (39).

To quantify the daily human mobility effects on the COVID-19 epidemic in all 219 TPUs, daily traffic flow data covering all Hong Kong's strategic routes between and including the entirety of December 2021 to February 2022, were used in this study (40). The COVID-19 vaccines used in Hong Kong were the Sinovac Vaccines and BioNTech Vaccines. In order to measure the impact of full and booster vaccinations on the COVID-19 epidemic, Hong Kong's daily vaccination rates were used from February 22nd, 2021, to February 4th, 2022 (23) (Figure 1). The vaccination effectiveness of BioNTech and Sinovac against symptomatic diseases for Omicron and Delta was determined, based on previous studies (33, 41–43) (Supplementary Table 1). In addition, the daily COVID-19 effective reproductive number R was obtained from December 2021 to February 2022 from reports by The University of Hong Kong (44).

### An enhanced urban-community-level WKDE model for predicting the onset risk of COVID-19 symptoms

The SARS-CoV-2 has been found to have a high viral load and levels of transmissibility around the date of symptom onset (45, 46). Hence, it is necessary to adopt appropriate data-driven spatiotemporal models to dynamically and individually assess onset risk levels. Shi et al. developed an extended weighted kernel density estimation (WKDE) model (28–31). This model presents a retrospective analysis based on spatiotemporal information regarding onset cases, and presents an inference of the infection date of each onset case, and further infers the spatial distribution (i.e, the kernel density surface) of the infection risk to people by the onset cases at past dates, and finally to predict the distribution of onset risk at future dates (28–31). In addition, according to the transmission law of COVID-19 (i.e., mainly through direct, indirect, and close contact between people), data on the dynamic flow of people was introduced into the model to improve prediction accuracy (28–31). The improved model is an extended WKDE model (28–31).

As a further development of the original extended WKDE model, the urban-community-level WKDE model, proposed in this study, includes the following three steps (28–31):

a) Conducting a retrospective analysis of the historical existence likelihood of the infection in each community location in which an onset case had remained at the location and an onset case suffered by a visitor to the location;
b) Making inferences on the historical existence likelihood of the infection in the entire city;
c) Making predictions about the epidemic onset risk in the entire city on a given day in the near future.

The main difference between the urban-community-level WKDE model and the original extended WKDE model was that at step (b) of the model, the historical existence likelihood of an infection in a random location in the entire region was formulated as


(1) $$\begin{array}{l} P_{Infection}(S,ti)\;=\;n{\left(ti\right)}^{-1}\sum_{j=1}^{n(ti)}R(S,ti)\times (1-VE(S,ti)\;\times \;Vp(S,ti))\\ \;\times \;M_{Inter}-TPU(S,ti)\times M_{Inter}-TPU(S,ti)\\ \;\times \;P_{Infection}(Lj,ti)\times Kh(S-Lj) \end{array}$$

where P_*Infection*_(*S, t*_*i*_) is the probability of any individual infected with COVID-19 infecting others in a random location, *S*, in the city on day *t*_*i*_; R(S, t_i_) denotes the COVID-19 effective reproductive number in the city on day t_i;_ V_E_(S, t_i_) denotes the vaccine effectiveness against symptomatic diseases in the city on day t_i_ (6, 32–36); V_P_(S, t_i_) is the proportion of the population who have been fully vaccinated in the city on day t_i_; *L*_*j*_ is the *j*-th location among the province places where the onset cases resided; *P*_*Infection*_(*L, t*_*i*_) denotes the probability that one onset case was infected on day *t*_*i*_ in a location *L;* and *K*_*h*_(*S* – *L*_*j*_) denotes a Gaussian kernel between locations *S* and *L*_*j*_(28–31). The values of *P*_*Infection*_ (*L*_*j*_, *t*_*i*_), *K*_*h*_ (*S* – *L*_*j*_), and *h* were determined in earlier model procedures (28–31).

*M*_*intra*_*TPU*_(*S, t*_*i*_) denotes a human mobility factor within a TPU containing location *S* on day *t*_*i*_, calculated as follows (28–31):


(2) $$\begin{array}{r} M\mathrm{int}\text{ra}\text{\_}\text{TPU(S,ti)=}{\text{i}}^{-1}\sum_{k=1}^{i}Xk \end{array}$$

where *X*_*k*_ denotes the daily traffic flow within the TPU containing location *S* on day *t*_*k*_ prior to *t*_*i*_.

*M*_*interTPU*_(*S, t*_*i*_) denotes a human mobility factor from other TPUs to the TPU containing location *S*, calculated as follows (28–31):


(3) $$\begin{array}{r} M\mathrm{int}\text{er}\text{\_}\text{TPU(S,ti)=}{\text{i}}^{-1}\sum_{k=1}^{i}Yk \end{array}$$

where *Y*_*k*_ denotes the daily traffic flow from other TPUs to the TPU containing location *S* on day *t*_*k*_ prior to *t*_*i*_.

Finally, the predicted risk in each location was divided by the maximum predicted risk among all locations on a specific date and thereby standardized to a value between 0 and 1. Different levels of onset risk have been defined as follows: low onset risk (0–0.2), low-medium onset risk (0.2–0.4), medium onset risk (0.4–0.6), medium-high onset risk (0.6–0.8), and high onset risk (0.8–1). The reliability of the predicted COVID-19 onset risk was evaluated using its spatial significance, i.e., the percentage of symptom onset cases on a future date to be predicted that would occur in the areas with a predicted onset risk >0.8 (identified as onset hotspots) (28–31).

## Results

### Whole genome phylogenetic analysis

**Figure 2:**
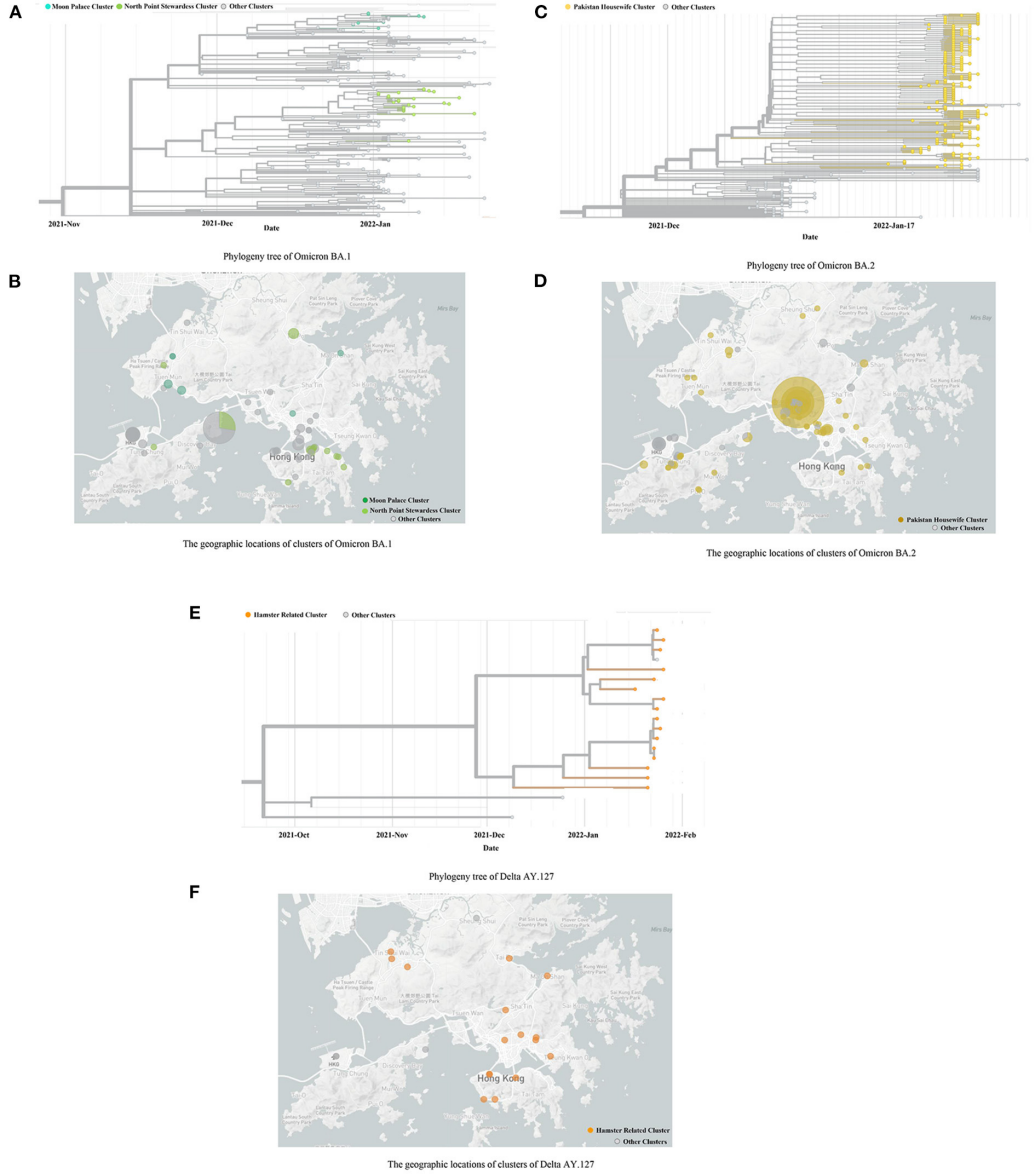
The Phylogeny trees and geographic locations of cases with Omicron BA.1, BA.2, and Delta AY.127. **(A,B)** indicates the Phylogeny trees and geographic locations of cases with Omicron BA.1. **(C,D)** indicates the Phylogeny trees and geographic locations of cases with Omicron BA.2. **(E,F)** indicates the Phylogeny trees and geographic locations of cases with Delta AY.127. **(A)** Phylogeny tree of Omicron BA.1. **(B)** The geographic locations of clusters of Omicron BA.1. **(C)** Phylogeny tree of Omicron BA.2. **(D)** The geographic locations of clusters of Omicron BA.2. **(E)** Phylogeny tree of Delta AY.127. **(F)** The geographic locations of clusters of Delta AY.127.

The whole genome sequencing results from, and including December 2021 to February 2022 have been used in this study. A total of 225 cases were identified as Omicron BA.1 from the period: December 2021 to January 2022. Of these cases, the majority (*n* = 191, 84.89%) were identified as imported cases and did not leak into the local community. Based on the phylogenetic analysis of the local cases, in late December 2021, two Omicron BA.1 strains were introduced to this community *via* aircrews exempt from quarantine. Each strain established discrete transmission chains: 8 local cases belonged to the Moon Palace cluster; 26 local cases belonged to the North Point cluster (Figure 2A). The locations of 34 local cases in these two clusters, are shown separately in Figure 2B.

A total of 329 cases was identified as Omicron BA.2, in which 231 were local cases. The source was thought to be a Pakistan housewife infected by another returnee living in an adjacent room of the quarantine hotel. This particular woman further infected nine family members, following her completion of the compulsory quarantine period. The family cluster eventually sparked the largest scale of COVID-19 epidemic in Hong Kong (Figure 2C). The locations of the premises which suffered clusters of 329 cases are shown in Figure 2D.

In addition to the Omicron variant, a total of 16 local cases was genotyped as Delta AY.127 (Figure 2E). These cases were attributed as hamster-to-human transmission and occurred owing to evidence of the variant within pet shops (10). The locations of the premises visited by the 16 local cases are shown in Figure 2F.

### How Omicron BA.1, BA.2, and Delta AY.127 spread spatiotemporally

**Figure 3:**
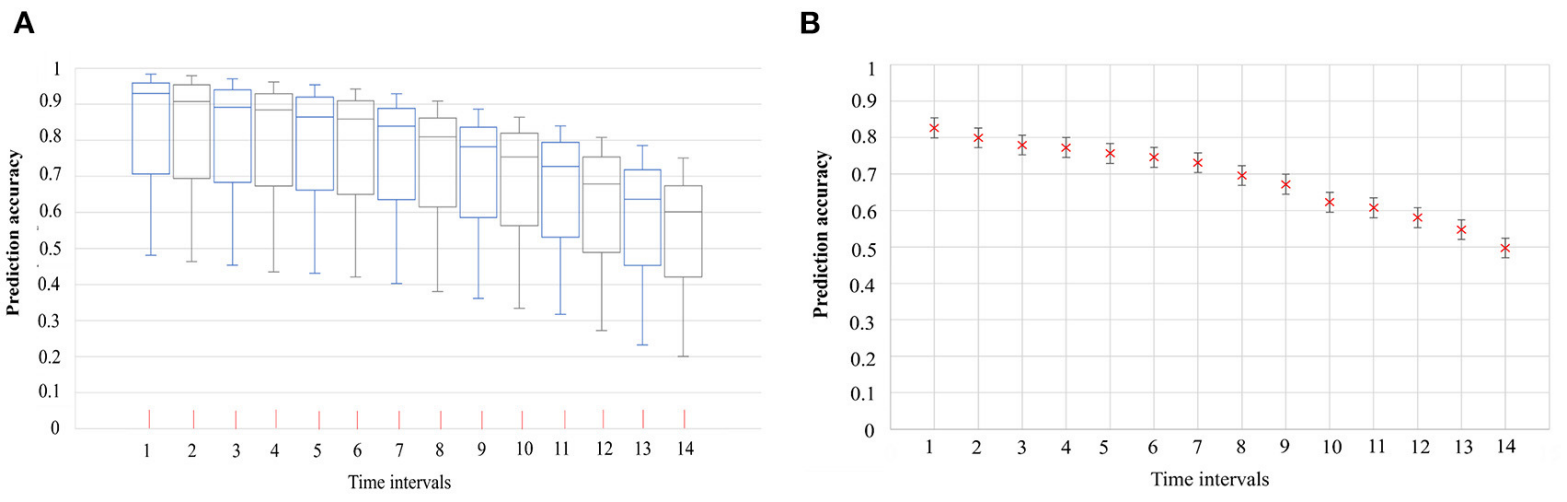
The accuracy of the predicted risk of COVID-19 symptom onset by urban-community-level WKDE models, and 95% confidence interval of prediction accuracy (28–31). **(A)** Accuracy of the predicted risk of COVID-19 symptom onset by urban-community-level WKDE models. The predicted onset risk is a normalized value of between 0 and 1, hence, indicating risk relative to the highest predicted risk among all locations, on the date for which the risk of symptom onset is predicted, hereafter termed “the prediction date”. The prediction accuracy is defined as the percentage of onset cases in those areas for which the predicted onset risk was higher than 0.8 on the prediction date. The time interval denotes the period between the base date (The base date refers to the date on which the onset risk for the next 14 days is predicted) and the date of prediction. The horizontal line in the box denotes the median, while the lower and upper edges of the box represent the respective first and third quartiles. The lines emanating from the box upwards and downwards represent the respective maximum and minimum values. **(B)** 95% confidence interval of the mean accuracy of the predicted risk of COVID-19 symptom onset by urban-community-level WKDE models.

The COVID-19 symptom onset risk in 291 TPUs in Hong Kong, during the early 20 days of the emergence and spread of Omicron BA.1, BA.2, Delta AY.127, was first predicted and further analyzed using the urban-community-level WKDE model. (i) 34 local Omicron BA.1 cases, (ii) 231 local Omicron BA.2 cases, and (iii) 16 local Delta AY.127 cases, with spatiotemporal information from Hong Kong during the period from December 26th, 2021, to February 4th, 2022, were used in the model. The prediction accuracy of the urban-community-level WKDE model was over 85% for symptom onset risk during the following seven days (28–31) (Figure 3). Such an ‘outlier-performance' should be attributed to the incorporation of (i) Locations with case resided/visited, (ii) locations with positive sewage testing results, (iii) time-varying vaccination rates, and vaccination efficiency.

**Figure 4:**
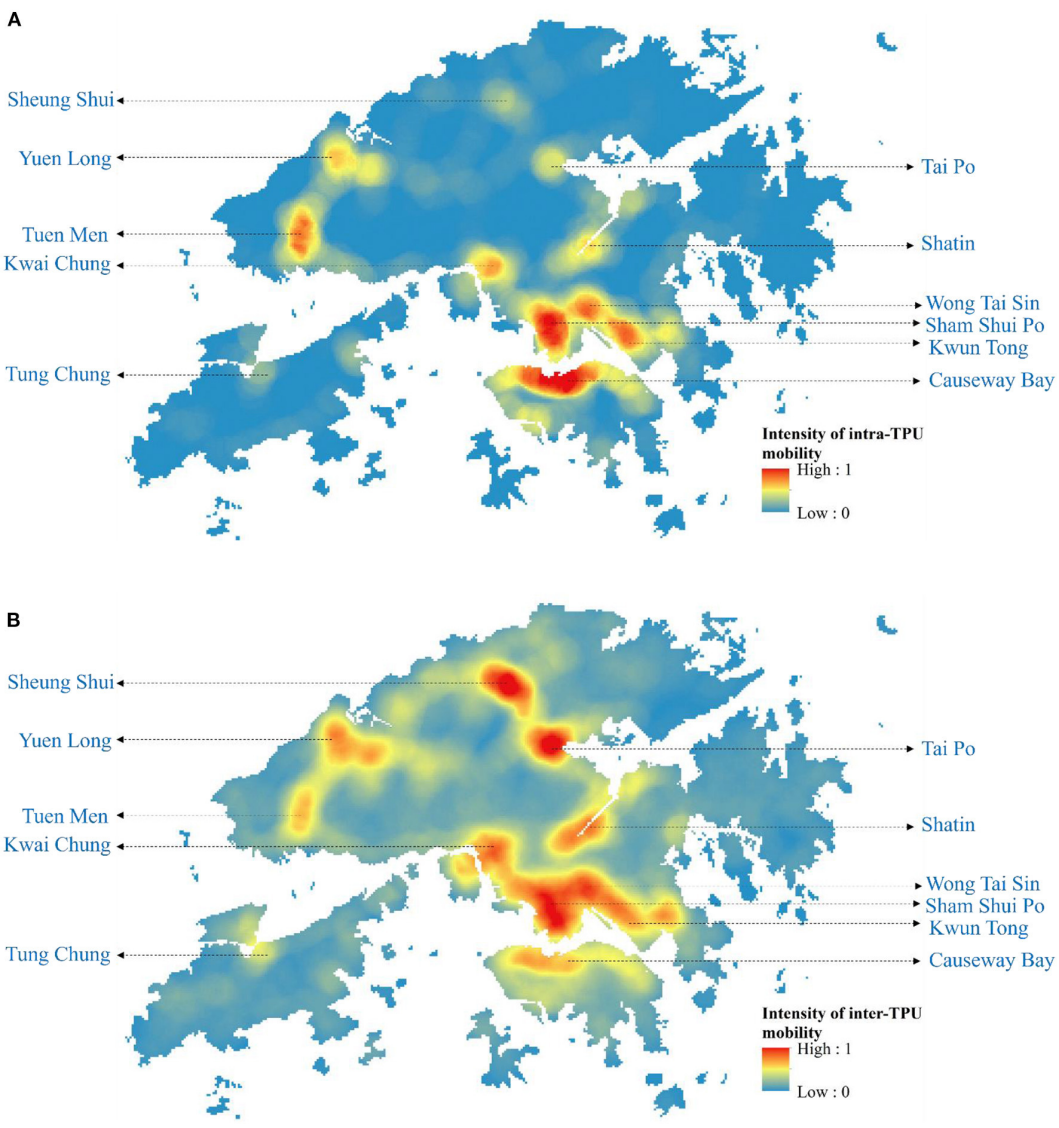
The intensity of intra-TPU and inter-TPU mobility of Hong Kong from December 2021 to February 2022. **(A)** The intensity of intra-TPU mobility of Hong Kong. **(B)** The intensity of inter-TPU mobility of Hong Kong.

**Figure 5:**
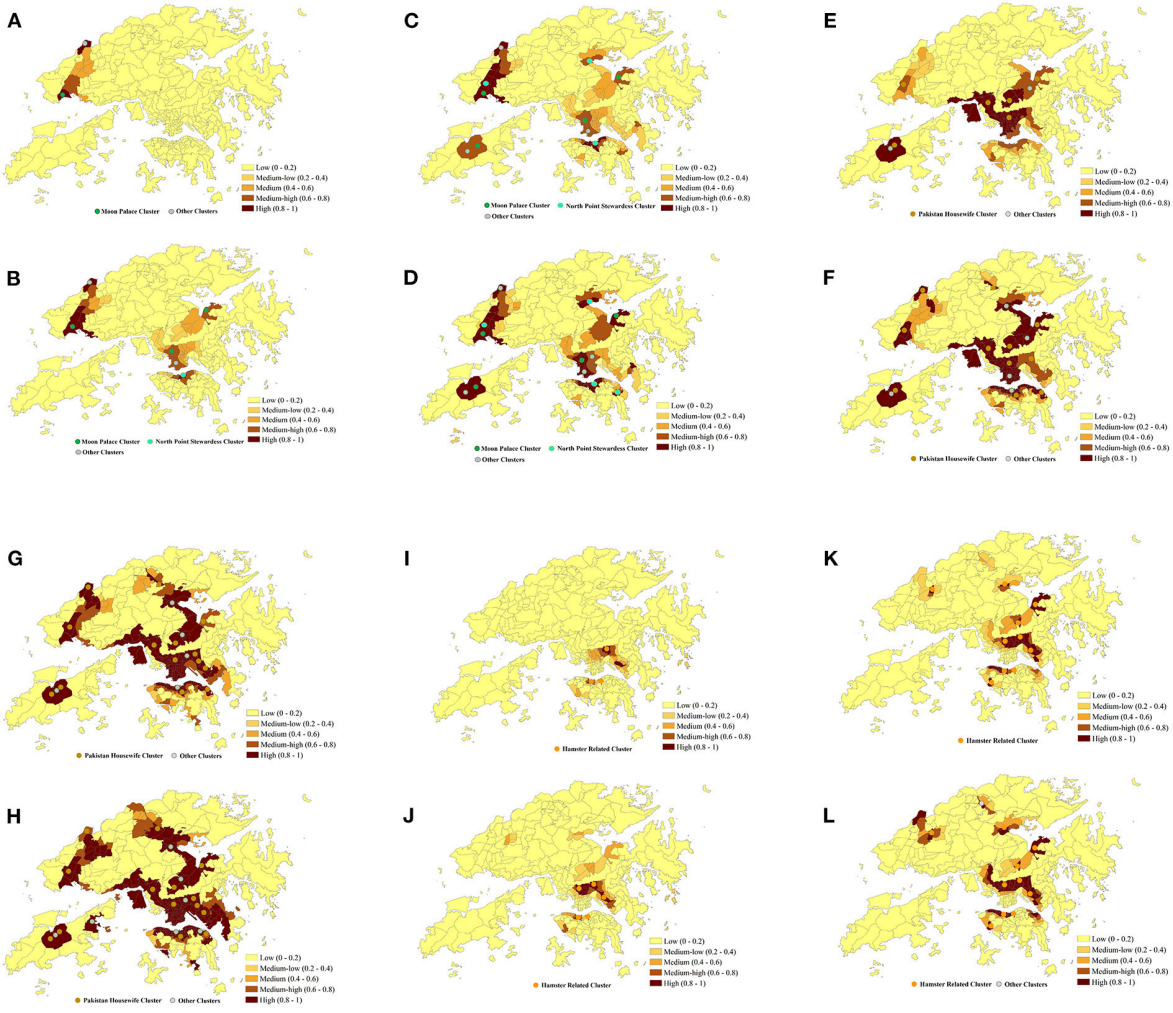
Predicted risk of COVID-19 symptom onset across 291 TPUs in Hong Kong within 20 days after Omicron BA.1, BA.2, and Delta AY.127 **(A–L)**. The predicted COVID-19 symptoms onset risk was generated using the urban-community-level WKDE model. **(A)** 5th day after Omicron BA.1 entered the community. **(B)** 10th day after Omicron BA.1 entered the community. **(C)** 15th day after Omicron BA.1 entered the community. **(D)** 20th day after Omicron BA.1 entered the community. **(E)** 20th day after Omicron BA.2 entered the community. **(F)** 10th day after Omicron BA.2 entered the community. **(G)** 15th day after Omicron BA.2 entered the community. **(H)** 20th day after Omicron BA.2 entered the community. **(I)** 5th day after Delta AY.127 entered the community. **(J)** 10th day after Delta AY.127 entered the community. **(K)** 15th day after Delta AY.127 entered the community. **(L)** 20th day after Delta AY.127 entered the community.

The emergence and spread of Omicron BA.1, BA.2, and Delta AY.127 during the first 20 days after these sub-linages entered the community could be described by the spatiotemporal variation of the predicted risk of COVID-19 symptom onset and the intensity of intra-TPU mobility (i.e., the traffic flow within the community) and inter-TPU mobility (i.e., the traffic flow across communities) as follows (Figure 4). Starting from Omicron BA.1 entering the community in Hong Kong, it can be seen that the TPUs in Tuen Mun, in which the related cases first appeared, started to reach high and medium-high onset risk level. Since then, due to the high intra-TPU human mobility in Tuen Mun, the number of high-risk TPUs in Tuen Mun continued to expand (Figures 4, 5A). At the same time, Omicron BA.1 spread to other distant TPUs with a similar high human mobility to that in Tuen Mun, such as TPUs in North Point, Sham Shui Po, Tai Po, and Sha Tin (Figures 4, 5B,C). These TPUs also became or at high or medium-high onset risk (Figures 5B,C). By the 20th day after Omicron BA.1 entered the community, there were 53 TPUs at high onset risk (Figure 5D), and involving 2,191,586 people (Table 1). In addition, the areas around the above, with high population mobility were also at medium or medium-low onset risk (Figures 4, 5D).

**Table 1:** Number of people living in high-onset-risk communities during the spread of Omicron BA.1, BA.2 and Delta AY.127 in Hong Kong.

	**5th day**	**10th day**	**15th day**	**20th day**
Omicron BA.1	385,242	666,544	1,579,093	2,191,586
Omicron BA.2	2,165,027	3,710,438	4,871,710	5,973,829
Delta AY.127	47,441	384,156	597,313	2,165,027

Omicron BA.2 also spread from the TPUs in Kwai Chung to TPUs with high population mobility within the human mobility network (Figures 4, 5E–H). The speed of spatial spread of Omicron BA.2 was obviously faster than that of BA.1. When Omicron BA.2 entered the community on the 10th day, almost all TPUs of high human mobility were at high onset risk, such areas included Sham Shui Po, Tuen Mun, Shatin, Causeway Bay, Tai Po, Yuen Long, Sheung Shui (Figures 4, 5G). TPUs in high-risk areas were 7.83 times more likely than in the period of the BA.1 spread. In fact, 3,043,894 more people were at high-onset-risk TPUs (Table 1). By the 20th day after Omicron BA.2 entered the community, other TPUs around these high-mobility TPUs were founded to be also at high onset risk (Figure 5H), making a final total of 154 (Figure 5H). There were 3,782,243 more people in the high-risk area relative to the same period of the Omicron BA.1 spread (Table 1).

The spread speed of the hamster-related Delta AY.127, was slower than that of both Omicron BA.1 and BA.2, despite its spread throughout the human mobility network (Figures 5I–K). On the 20th day when Delta AY.127 entered the community, only 43 TPUs of high population mobility in Wong Tai Sin, Shatin, Kwun Tong, Causeway Bay were at high onset risk (Figure 5L). On the 20th day of the spread of Omicron BA.1 and BA.2, TPUs at high-onset-risk were respectively, 1.23 times and 3.55 times greater than that of Delta AY.127 during the same period. There were also 3,808,802 and 26,559 more people in high-onset-risk TPUs with the spread of Omicron BA.1 and BA.2 than with that with Delta AY.127 in the same period (Table 1). The impact of Delta AY.127 on those TPUs which surrounded these particular TPUs with high human mobility, appears to have been relatively limited.

In addition, during the 20 days of the spread of Omicron BA.1, BA.2, Delta AY.127, and according to the exploration of the correlation between the intensity of intra-TPU/inter-TPU mobility and the symptom onset risk (Table 2), it can be found that the intensity of population mobility within the community promotes spread of these variants to adjacent areas. For example, on the 20th day after the start of the Omicron BA.2 spread, the R^2^ between the intensity of intra-TPU mobility and the symptom onset risk reached 0.87, this being 0.0344 and 0.0967 higher than that of Omicron BA.1, Delta AY.127 (Table 2). Similarly, the intensity of population mobility across communities promotes the spread of variants to other communities with closely-connected traffic. For example, on the 20th day after the start of Omicron BA.2 spread, the R^2^ between the intensity of inter-TPU mobility and the symptom onset was 0.0394 and 0.1188 higher than that for Omicron BA.1, Delta AY.127 (Table 2). The effect of intra-TPU/inter-TPU human mobility on the spatial spread of Omicron BA.1, BA.2, and Delta AY.127 became more and more significant over time.

**Table 2:** The correlation between the symptom onset risk and the intensity of intra-TPU and inter-TPU mobility during the spread of Omicron BA.1, BA.2 and Delta AY.127 in Hong Kong.

**Sublineages**	**Human mobility**	***R*^2^ (5th day)**	***R*^2^ (10th day)**	***R*^2^ (15th day)**	***R*^2^ (20th day)**
Omicron BA.1	With the intensity of intra-TPU mobility	0.7254	0.7601	0.7979	0.8370
	With the intensity of inter-TPU mobility	0.6756	0.7424	0.7758	0.7957
Omicron BA.2	With the intensity of intra-TPU mobility	0.7649	0.8068	0.8397	0.8714
	With the intensity of inter-TPU mobility	0.7154	0.7791	0.8118	0.8351
Delta AY.127	With the intensity of intra-TPU mobility	0.6427	0.7093	0.7486	0.7747
	With the intensity of inter-TPU mobility	0.5815	0.6566	0.6744	0.7163

**Figure 6:**
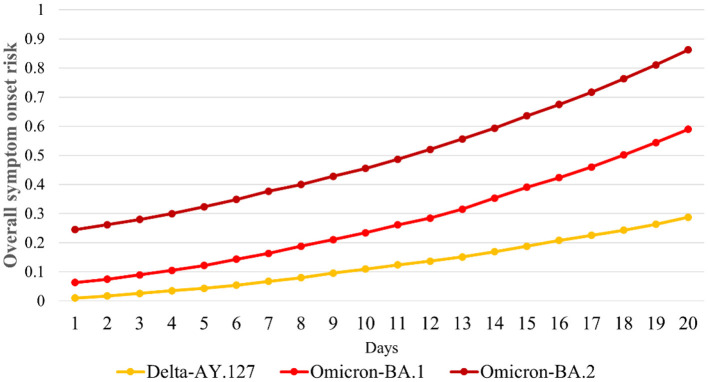
Daily overall risk of COVID-19 symptom onset in Hong Kong within 20 days after Omicron BA.1, BA.2, and Delta AY.127.

We further compared the variations in the overall symptom onset risk of 291 TPUs within 20 days of Omicron BA.1, BA.2, and Delta AY.127 entering the community. The overall onset risk increased from 0.06 to 0.58 within 20 days of Omicron BA.1 spread (Figure 6). The overall risk of disease increased from 0.25 to 0.86 within 20 days of the spread of Omicron BA.2, which was a 1.16-fold increase over the risk of spread of Omicron BA.1 (Figure 6). By the 20th day of Omicron BA.2 entering the community, the overall symptom onset risk in Hong Kong was already at the high-onset-risk level. In contrast, the overall risk increases in the first 20 days of Delta AY.127 spread decreased by 58 and 78% relative to the Omicron BA.1 and BA.2 periods, respectively (Figure 6). By the 20th day of Delta AY.127 entering the community, the overall symptom onset risk in Hong Kong was still at low-medium onset risk level (Figure 6).

### The spatiotemporal symptom onset risk with Omicron BA.1, BA.2, and Delta AY.127 under different vaccination scenarios

According to the variation of the overall onset risk in 291 TPUs in Hong Kong, the overall symptom onset risk (the mean value of the symptom onset risk in 291 TPUs) increased by 0.53, 0.62, and 0.28 within 20 days of Omicron BA.1, BA.2, and Delta AY.127 entering the community (Figure 6). The overall onset risk of Omicron BA.1 and BA.2 on the 20th day after entering the community even reached the medium onset risk level and the high-onset-risk level, respectively. Previous studies have found that vaccination, especially booster vaccination, has significantly improved protection against the symptomatic infection of Omicron and Delta. However, only limited full and booster vaccine occurred increased in Hong Kong because Sinovac and BioNTech vaccination appears to have limited effect in reducing overall onset risk. Thus, simulations were made on how to strengthen vaccination so as to further decrease the symptom onset risk of Omicron BA.1, BA.2, and Delta AY.127 in Hong Kong, in particular, the booster vaccination rates. The simulation was conducted in (i) the current full and booster vaccination rate, (ii) 5 times the full vaccination rate and the current booster vaccination rate, (iii) 5 times the full and booster vaccination rate, (iv) 10 times the full vaccination rate and the current booster vaccination rate, and (v) 10 times the full and booster vaccination rate. During the simulation process, the vaccine effectiveness of Sinovac and BioNTech Vaccine, which decreases over time, were also taken into account.

**Figure 7:**
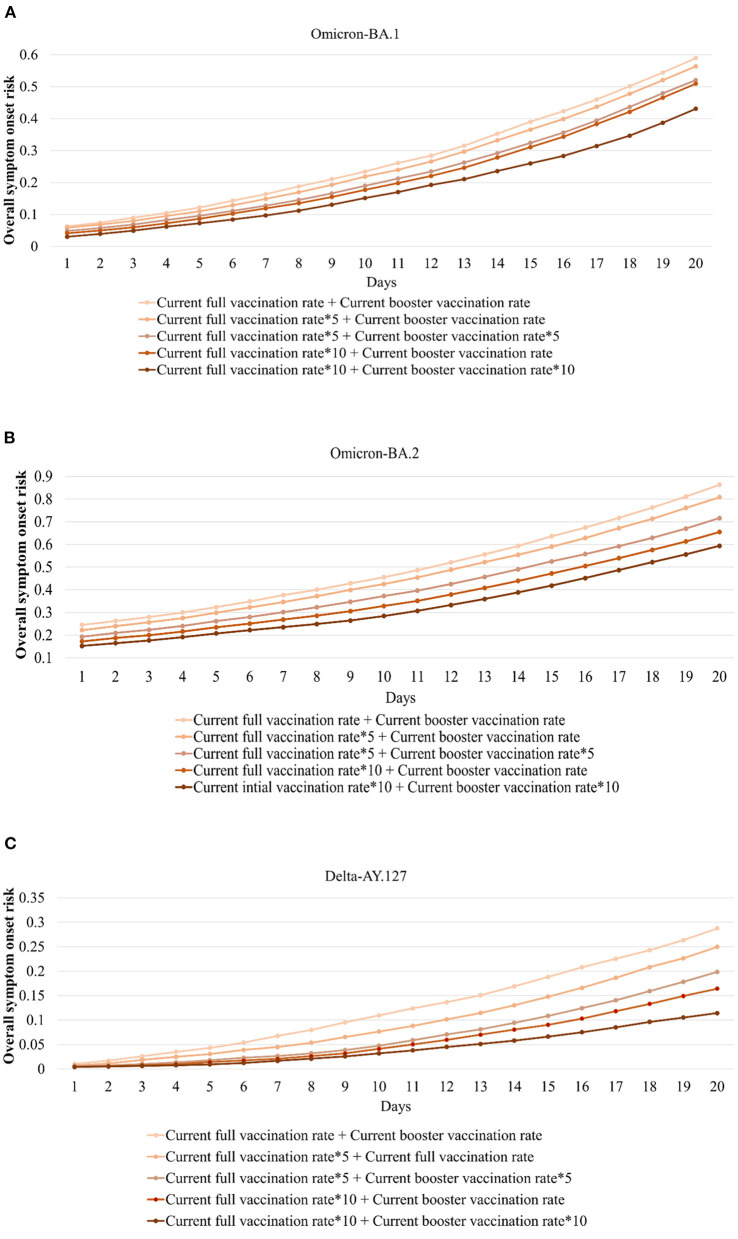
Daily overall symptom onset risk with the full and booster vaccination rate increased by 5 or 10 times during the 20 days when Omicron BA.1, BA.2, and Delta AY.127 entered the community. **(A)** indicates daily overall symptom onset risk of Omicron BA.1. **(B)** indicates daily overall symptom onset risk of Omicron BA.2. **(C)** indicates daily overall symptom onset risk of Delta AY.127.

The temporal variation of the daily overall onset risk values within 20 days of Omicron BA.1, BA.2, and Delta AY.127 entering the community in Hong Kong under the above five scenarios, were explored to reflect the effects of improved full and booster vaccination rate (Figure 7). Within 20 days of Omicron BA.1 entering the community, the full vaccination rate was increased by 5 times (an increase of 175,000 people receiving the Sinovac vaccine, 170,000 people receiving the BioNTech vaccine) or 10 times (an increase of 394,000 people receiving the Sinovac vaccine, 383,000 people receiving the BioNTech vaccine), the overall symptom onset risk decreased by an average of 7.89 and 24.22% respectively (Figure 7A). On this basis, when the booster vaccination rate was increased, by 5 times (an increase of 323,000 people receiving the Sinovac vaccine, 444,000 people receiving the BioNTech vaccine) and 10 times (an increase of 726,000 people receiving the Sinovac vaccine, 1,000,000 people receiving the BioNTech vaccine), the overall symptom onset risk was further reduced by an average of 12.25 and 16.90% (Figure 7A). Compared with Omicron BA.1, the effect of increased vaccination rate on the reduction of overall symptom onset risk was relatively less within 20 days of Omicron BA.2 entering the community. When the full vaccination rate was increased by 5 times (an increase of 165,000 people receiving the Sinovac vaccine, 278,000 people receiving the BioNTech vaccine) and 10 times (an increase of 371,000 people receiving the Sinovac vaccine, 627,000 people receiving the BioNTech vaccine), the overall onset risk was reduced by 4.16 and 21.01% respectively (Figure 7B). When the booster vaccination rate was also increased by 5 times (an increase of 429,000 people receiving the Sinovac vaccine, 967,000 people receiving the BioNTech vaccine) and 10 times (an increase of 965,000 people receiving the Sinovac vaccine, 2176,000 people receiving the BioNTech vaccine), the overall onset risk was further reduced by 7.36 and 11.49% (Figure 7B). An increased vaccination rate was also effective in reducing the risk of symptom onset due to the hamster-related Delta AY.127. When the full vaccination rate was increased by 5 times (an increase of 148,000 people receiving the Sinovac vaccine, 266,000 people receiving the BioNTech vaccine) and 10 times (an increase of 334,000 people receiving the Sinovac vaccine, 600,000 people receiving the BioNTech vaccine), the overall onset risk was reduced by 25.30 and 58.54% (Figure 7C). When the booster vaccination rate was also increased by 5 times (an increase of 539,000 people receiving the Sinovac vaccine, 1,080,000 people receiving the BioNTech vaccine) and 10 times (an increase of 1,210,000 people receiving the Sinovac vaccine, 2,450,000 people receiving the BioNTech vaccine), the overall onset risk was further reduced by 32.37 and 23.63% (Figure 7C).

**Figure 8:**
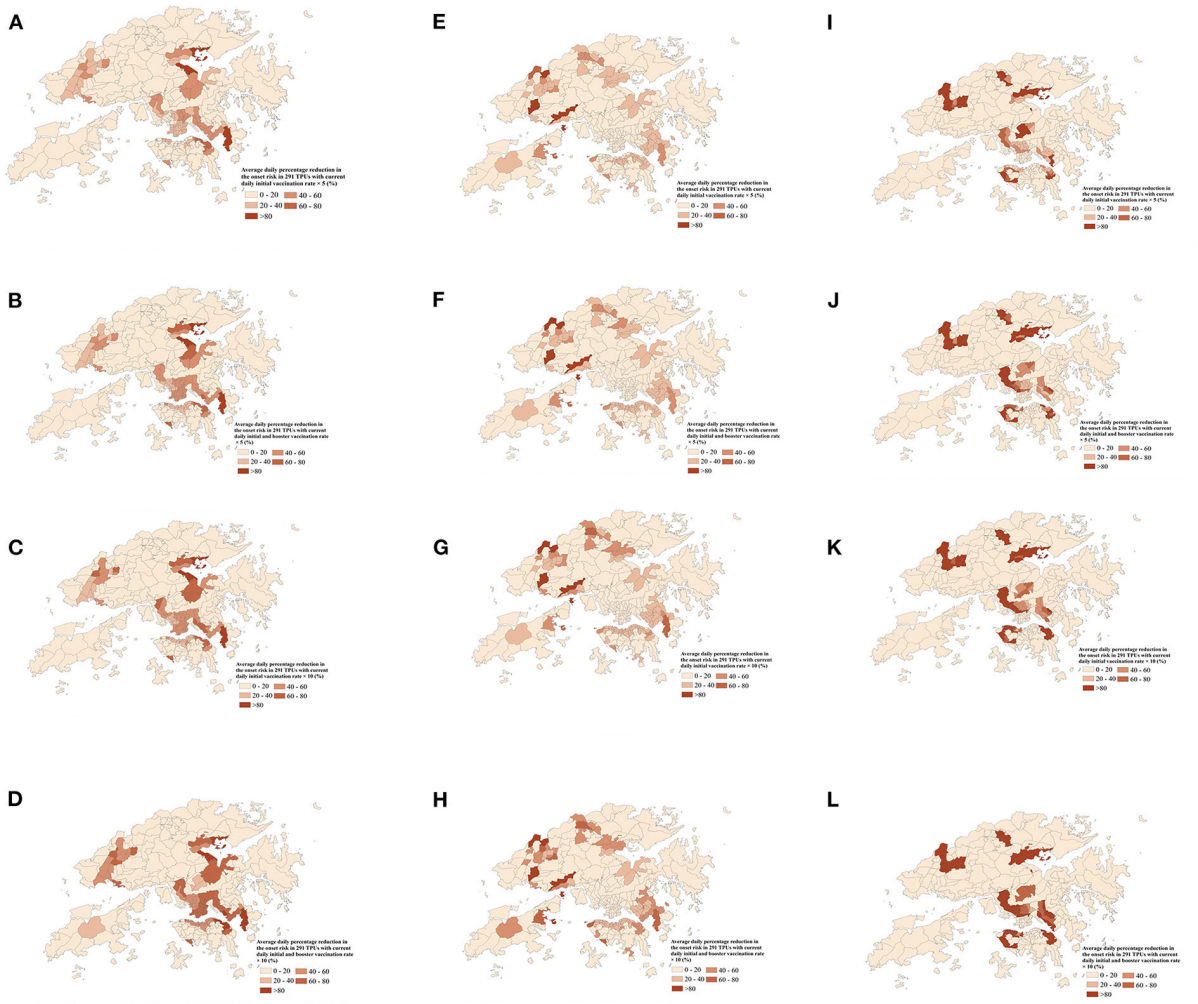
The average daily percentage reduction in the symptom onset risk in 291 TPUs of Hong Kong with 5 times or 10 times the current daily full and booster vaccination rate, compared with the current daily full and booster vaccination rate. **(A-D)** indicates the average daily percentage reduction in the symptom onset risk of Omicron BA.1 in 291 TPUs. **(E-H)** indicates the average daily percentage reduction in the symptom onset risk of Omicron BA.2 in 291 TPUs. **(I-L)** indicates the average daily percentage reduction in the symptom onset risk of Delta AY.127 in 291 TPUs. **(A)** Average daily percentage reduction in the onset risk in 291 TPUs with 5 times current daily full vaccination rate—Omicron BA.1. **(B)** Average daily percentage reduction in the onset risk in 291 TPUs with 5 times current daily full and booster vaccination rate—Omicron BA.1. **(C)** Average daily percentage reduction in the onset risk in 291 TPUs with 10 times current daily full vaccination rate—Omicron BA.1. **(D)** Average daily percentage reduction in the onset risk in 291 TPUs with 10 times current daily full and booster vaccination rate—Omicron BA.1. **(E)** Average daily percentage reduction in the onset risk in 291 TPUs with 5 times current daily full vaccination rate—Omicron BA.2. **(F)** Average daily percentage reduction in the onset risk in 291 TPUs with 5 times current daily full and booster vaccination rate—Omicron BA.2. **(G)** Average daily percentage reduction in the onset risk in 291 TPUs with 10 times current daily full vaccination rate—Omicron BA.2. **(H)** Average daily percentage reduction in the onset risk in 291 TPUs with 10 times current daily full and booster vaccination rate—Omicron BA.2. **(I)** Average daily percentage reduction in the onset risk in 291 TPUs with 5 times current daily full vaccination rate—Delta AY.127. **(J)** Average daily percentage reduction in the onset risk in 291 TPUs with 5 times current daily full and booster vaccination rate—Delta AY.127. **(K)** Average daily percentage reduction in the onset risk in 291 TPUs with 10 times current daily full vaccination rate—Delta AY.127. **(L)** Average daily percentage reduction in the onset risk in 291 TPUs with 10 times current daily full and booster vaccination rate—Delta AY.127.

Furthermore, the onset risk in all 291 TPUs which had 5 to 10 times the current daily full and booster vaccination rate, on the same date, was obviously lower than the risk with the current daily full and booster vaccination rate (Figure 8). When full vaccination rate was increased by 5 times and 10 times, the symptom onset risk in each TPU caused by Omicron BA.1 transmission decreased by 21.83 and 32.76% (Figures 8A–C), respectively, especially for medium and medium-low onset risk TPUs around areas of high human mobility. Over 91.79% of medium and medium-low onset risk TPUs had a maximum 90.15% reduction regarding symptom onset risk. When the booster vaccination rate increased by 5 or 10 times, the symptom onset risk would decrease by 32.45 and 43.12%, especially for these medium-high onset risk TPUs around areas of high human mobility (Figures 8B–D). Over 97.82% of the medium and medium-high onset risk TPUs had a maximum 56.48% reduction in symptom onset risk. Likewise, the increased vaccination rate had a clear effect on reducing the risk of Omicron BA.2 (Figures 8E–H). When the full and booster vaccination rate was increased 10 times, the symptom onset risk in each TPU was reduced by an average of 25.86% (Figure 8G). However, compared with Omicron BA.1, the effect of increasing the full and booster vaccination rate on the reduction of the symptom onset risk of Omicron BA.2 appears to be mainly for TPUs of medium-low and medium onset risk, respectively. The symptom onset risk in these TPUs was reduced by up to 82.34% (Figures 8B–D). In addition, increasing vaccination rates had the most significant effect on reducing the risk of hamster-associated Delta AY.127 (Figures 8I-L). When the full and booster vaccination rate was increased by 10, the symptom onset risk in each TPU was reduced by an average of 92.13% (Figure 8L). in fact, more than 80.97% of TPUs at high onset risk had an average of 83.21% lower symptom onset risk.

## Discussion

Currently, with the widespread vaccination (47, 48) and the results of previous infections (49, 50), the COVID-19 immunity barrier in some regions was gradually established. Even for the Omicron variant, previous studies have shown that the COVID-19 vaccine is effective in preventing serious illness and death caused by Omicron (51–53). The pandemic, hence, entered another phase. As regards the advent of medically critical illnesses, one of the results is the necessity to minimize and prevent undue stress on the health care sector and thereby produce adequate care for patients, has become one of the keys to the development of epidemic prevention efforts (54). As a result, major countries in the world have begun to focus on symptomatic cases, especially severe cases, in epidemiological surveillance (17, 55). For example, the US CDC requires that case investigation should focus on symptomatic cases and assessing COVID-19 community levels (16). In Hong Kong, which when at the peak of the epidemic in March of 2022, due to the surge in cases, in order to avoid the overuse of medical resources, the focus of epidemic prevention and control has also been on the monitoring and treatment of symptomatic cases, especially severe cases. Hence, this study provides an analysis and comparison of the spatiotemporal symptom onset risks associated with Omicron BA.1, BA.2, and hamster-related Delta AY.127 during their early spread at 291 TPUs in Hong Kong, to support Hong Kong and the world to better deal with these new variants. Of importance, in this respect, is that, based on the SARS-CoV-2 sequences and the epidemiological link of symptomatic cases determined by whole-genome sequencing, this study used the enhanced urban-community-level WKDE model to enable the prediction of the symptom onset risk with SARS-CoV-2 variants. Furthermore, the spatiotemporal spread of Omicron BA.1, BA.2, and hamster-related Delta AY.127 under different full and booster vaccination scenarios have also been analyzed.

With the use of whole genome sequencing technology, the SARS-CoV-2 sequences and related cluster of symptomatic cases, within 20 days after Omicron BA.1, BA.2, and Delta AY.127 entered the community, were identified. Timely use of whole genome sequencing can greatly facilitate outbreak investigation and understanding of the cryptic chain of transmission, to further help to explore the spatiotemporal symptom onset risk. Based on i) locations with symptomatic cases, ii) locations with positive sewage testing results, and iii) time-varying vaccination rate and vaccination efficiency to strengthen the urban-community-level WKDE model, the model achieved a high accuracy with more than 85% in the onset risk prediction of future 7 days.

Thus, the spatiotemporal variation of the symptom onset risk with Omicron BA.1, BA.2, Delta AY.127 during the associated first 20 days of transmission in Hong Kong could be analyzed. The results are shown in this study:

i) The spatial transmissibility ability of Omicron BA.2 is obviously stronger than that of Omicron BA.1 and Delta AY.127. By the 20th day after Omicron BA.2 entered the community, a total of 154 TPUs were at high onset risk, which were 2.91 and 3.56 times faster than the spread of Omicron BA.1 and Delta AY.127. There were 3,782,243 and 3,808,802 more people in the high-risk area communities than during the same period of spread of Omicron BA.1 and Delta AY.127. Conversely, the spatiotemporal spread of hamster-relate Delta AY.127 was weaker than that of Omicron BA.1 and BA.2. On the 20th day when Delta AY.127 entered the community, only 43 TPUs of high population mobility were at high-onset-risk, which were 0.28 and 0.81 times of that in the same period as Omicron BA.2, BA.1 spread. There were also 3,808,802 and 26,559 fewer people in high-onset-risk TPUs than that with Omicron BA.2, BA.1 in the same period.
ii) Omicron BA.1, BA.2, and Delta AY.127 all started to emerge at the high human mobility community level linked with the traffic flow network. Afterwards, the intensity of human mobility within the community continued to promote the spread of the above SARS-CoV-2 variants to adjacent areas. The intensity of human mobility across communities further continued to promote the spread of SARS-CoV-2 variants to other communities of high human mobility levels, further away. Among these, the effect of inter-community and cross-community human mobility on the enhancement of the spatiotemporal spread of Omicron BA.2 was much more significant than that of Omicron BA.1 and Delta AY.127. This enhancement accumulated over time.
iii) The spread of Omicron BA.1, BA.2, and Delta AY.127 also had a certain impact on the temporal variation of the overall symptom onset risk in Hong Kong over time, but the impact intensity was different. The overall symptom onset risk increased from 0.25 to 0.86 within 20 days of the spread of Omicron BA.2, which was 1.16 times increase during the spread of Omicron BA.1. The overall symptom onset risk in Hong Kong was already at a high-onset-risk level. In contrast, by the 20th day of Delta AY.127 entering the community, the overall symptom onset risk in Hong Kong increased only to the low-medium risk level. The overall risk increases within 20 days of Delta AY.127 spread decreased by 58% and 78% relative to the respective Omicron BA.1 and BA.2 periods.

Through the simulation and comparison of the spatiotemporal symptom onset risk with Omicron BA.1, BA.2, and Delta AY.127 under different scenarios with different levels of full and booster vaccination rates, this study also provides a scientific reference for areas attacked by SARS-CoV-2 variants. The results show that:

1) When the full vaccination rate is increased, the overall symptom onset risk with Omicron BA. 1, BA.2, and Delta AY.127 in Hong Kong is effectively reduced. If the booster vaccination rate is further increased, the overall symptom onset risk can be likewise, further reduced. Within 20 days of Omicron BA.1 entering the community, when the full vaccination rate was increased by 394,000 people receiving the Sinovac vaccine and 383,000 people receiving the BioNTech vaccine, the overall symptom onset risk decreased by an average of 24.22%. On this basis, when the booster vaccination rate was increased by 726,000 people receiving the Sinovac vaccine, and 1,000,000 people receiving the BioNTech vaccine, the overall symptom onset risk was further reduced by an average of 16.90%. Compared with Omicron BA.1, the reduction effect of increased vaccination rate on the overall symptom onset risk of Omicron BA.2 was lower. When the full vaccination rate was increased by 371,000 people receiving the Sinovac vaccine, and 627,000 people receiving the BioNTech, the overall onset risk was only reduced by 4.16 and 21.01%. When the booster vaccination rate was further increased by 965,000 people receiving the Sinovac vaccine, and 2,176,000 people receiving the BioNTech vaccine, the overall onset risk was further reduced by 7.36 and 11.49%. Increased vaccination rates were most effective in reducing the risk of symptom onset due to hamster-related Delta AY.127. When the full vaccination rate was increased by 334,000 people receiving the Sinovac vaccine, and 600,000 people receiving the BioNTech vaccine, the overall onset risk was reduced by 25.30 and 58.54%. When the booster vaccination rate was increased by 1210,000 people receiving the Sinovac vaccine, and 2,450,000 people receiving the BioNTech vaccine, the overall onset risk was further reduced by 32.37 and 23.63%.
2) The increased full and booster vaccination rates can effectively reduce the symptom onset risk for each TPU. The above reduction effect is different for Omicron BA.1, BA.2, and Delta AY.127. For Omicron BA.1, when the full vaccination rate was increased by 10 times, the symptom onset risk in each TPU decreased by 32.76%, especially for medium and medium-low onset risk TPUs around areas of high human mobility. When the booster vaccination rate was also increased by 10 times, the symptom onset risk decreased by 43.12%, especially for the medium-high onset risk TPUs, around areas of high human mobility. But for Omicron BA.2, when the full and booster vaccination rates were increased by 10 times, although onset risk in each TPU was reduced by an average of 25.86%, the reduction effect seemed to be mainly for TPUs of medium-low and medium onset risk. The reduction effect of the symptom onset risk for TPUs originally at high and medium-high risk was more limited. Additionally, increasing vaccination rates could have the most significant effect on reducing the risk of hamster-associated Delta AY.127. When the full and booster vaccination rates were increased 10 times, the simulated symptom onset risk in each TPU was reduced by an average of 92.13%, even for TPUs of high onset risk.

This current study has several limitations worthy of further examination. Firstly, the traffic flow data used in this study to measure daily human mobility is composed only of the official total population traffic flow on major arterial roads in Hong Kong. This restricts our exploration of COVID-19 symptom onset risk in different age groups and genders facing Omicron BA.1, BA.2, and Delta AY.127. Recently, we are applying to China Mobile Hong Kong for the main relevant mobile phone signaling data of different age groups to analyze their daily mobility, which has the potential to support the further exploration of COVID-19 symptom onset risk in people of different ages and genders. Secondly, lack of vaccine efficacy data related to sub-variants, the vaccination effectiveness against Omicron and Delta has been used in this study. However, the predictive performance of this proposed model can be even further improved if further sufficiently reliable vaccine effectiveness data against Omicron BA.1, BA.2, and Delta AY.127, especially for Sinovac vaccines, can be achieved. Importantly the acquisition of reliable effective vaccine data against Omicron BA.1, BA.2, and Delta AY.127 related to different age groups and genders, will further strengthen the additional study of COVID-19 symptom onset risk for different ages and different genders.

WHO requests countries and regions to continue to be vigilant, to monitor and report new sequences, as well as to conduct independent and comparative analyses of the different Omicron sub-lineages (2). Furthermore, WHO also encourages countries and regions to share available data on transmissibility and severity of variants, and their impact on diagnostics and vaccines (2). As the study has provided a comprehensive investigation about the spatiotemporal symptom onset risk of Omicron BA.1, BA.2, and Delta AY.127, we hope that this study can assist countries and regions to prevent the emergence of these variants on a strategic basis. Moreover, for countries and regions where the new SARS-CoV-2 variant Omicron has appeared, this study provides scientifically derived findings on the impact of the full and booster vaccination campaigns on reducing the symptomatic infection.

## Data availability statement

Publicly available datasets were analyzed in this study. The Whole Genome Sequencing data can be found at the Nextstrain repository: https://nextstrain.org/groups/ncovHK/ncov/Latest?c=transmission_alert&p%24=%24full; The real-time effective reproductive number data can be found at https://covid19.sph.hku.hk/; The vaccination data can be found at https://www.covidvaccine.gov.hk/en/dashboard. The traffic Data of Strategic/Major Roads can be found at https://data.gov.hk/en-data/dataset/hk-td-sm_4-traffic-data-strategic-major-roads.

## Author contributions

WS conceived and designed the study, interpreted the results, and helped develop the computation models, analyze the data, and write the manuscript. CT collected the data, developed the computation models, wrote the manuscript, and helped interpreted the results. GS collected the data, conducted the whole gene sequencing, and helped write the manuscript. AZ helped develop the computation models, analyze the data, and write the manuscript. ZS wrote the manuscript, helped collect the data, and analyze the data and interpret the results. All authors contributed to the article and approved the submitted version.

## Funding

This study was supported by National Key R&D Program of China (2019YFB2103102), Hong Kong Research Grants Council (C5079-21G), and Otto Poon Charitable Foundation Smart Cities Research Institute, The Hong Kong Polytechnic University (Work Program: CD03).

## Conflict of interest

The authors declare that the research was conducted in the absence of any commercial or financial relationships that could be construed as a potential conflict of interest.

## Publisher's note

All claims expressed in this article are solely those of the authors and do not necessarily represent those of their affiliated organizations, or those of the publisher, the editors and the reviewers. Any product that may be evaluated in this article, or claim that may be made by its manufacturer, is not guaranteed or endorsed by the publisher.
