# Supplementary material for: Understanding spatiotemporal symptom onset risk of Omicron BA.1, BA.2 and hamster-related Delta AY.127
Source: Front Public Health. 2022 Sep 16;10:978052. doi: 10.3389/fpubh.2022.978052 (PMC9523538; doi:10.3389/fpubh.2022.978052)
Supplement: Supplementary file 1 [file Table_1.DOCX]

| Virus | Vaccine | Weeks | Vaccine  Effectiveness (%)  -Omicron | Vaccine  Effectiveness (%)  -Delta |
| --- | --- | --- | --- | --- |
| BNT162b2 | Dose 1 | 4+ | 34.2 | 36.1 |
|  | Dose 2 | 2-9 | 88 | 88.2 |
|  |  | 10-14 | 48.5 | 77.7 |
|  |  | 15-19 | 34.1 | 72.2 |
|  |  | 20-24 | 36.6 | 64.8 |
|  |  | 25+ | 34.2 | 63.5 |
|  | Booster | 2+ | 75.5 | 92.6 |
| Sinovac | Dose 1 | 4+ | 0 | 29.9 |
|  | Dose 2 | 2-9 | 3 | 63.3 |
|  |  | 10-14 | 1 | 45.5 |
|  |  | 15-19 | 1 | 39.1 |
|  |  | 20-24 | 1 | 29.8 |
|  |  | 25+ | 1 | 22.2 |
|  | Booster | 2+ | 36 | 41.7 |

**Supplementary Table 1: Vaccine effectiveness (%) against symptomatic diseases by period after dose 1 and dose 2 for Omicron and Delta (1-4)**

**Reference**

1. D24H@HKSTP and HKU WHO Collaborating Centre on Infectious Disease Epidemiology and Modelling. Update report dated February 21, 2022. (2022). Available online at: https://www.med.hku.hk/en/news/press/-/media/575E4CA7F5584164B108386D3EF7A6E3.ashx (accessed February 1, 2022).

2. Andrews N, Stowe J, Kirsebom F, Toffa S, Rickeard T, Gallagher E, Gower C,

Kall M, Groves N, O’Connell A-M, et al. Covid-19 Vaccine Effectiveness against the Omicron (B.1.1.529) Variant. *N Engl J Med* (2022)1–15. doi: 10.1056/NEJMoa2119451

3. McMenamin ME, Nealon J, Lin Y, Wong JY, Cheung JK, Lau EHY, Wu P, Leung

GM, Cowling BJ. Vaccine effectiveness of one, two, and three doses of BNT162b2 and CoronaVac against COVID-19 in Hong Kong: a population-based observational study. Lancet Infect Dis. 2022 Jul 15:S1473-3099(22)00345-0. doi: 10.1016/S1473-3099(22)00345-0.

4. Wu D, Zhang Y, Tang L, et al. Effectiveness of Inactivated COVID-19 Vaccines Against Symptomatic, Pneumonia, and Severe Disease Caused by the Delta Variant: Real World Study and Evidence - China, 2021. China CDC Wkly. 4:57-65. doi: 10.46234/ccdcw2022.009.
